# Caregiver Perceptions and Motivation for Disclosing or Concealing the Diagnosis of HIV Infection to Children Receiving HIV Care in Mbarara, Uganda: A Qualitative Study

**DOI:** 10.1371/journal.pone.0093276

**Published:** 2014-03-25

**Authors:** Julius Kiwanuka, Edgar Mulogo, Jessica E. Haberer

**Affiliations:** 1 Department of Paediatrics and Child Health, Mbarara University of Science and Technology, Mbarara, Uganda; 2 Department of Community Health, Mbarara University of Science and Technology, Mbarara, Uganda; 3 Massachusetts General Hospital, Center for Global Health, Boston, Massachusetts, United States of America; 4 Harvard Medical School, Boston, Massachusetts, United States of America; University of Washingto, United States of America

## Abstract

**Background:**

Disclosure of the diagnosis of HIV to HIV-infected children is challenging for caregivers. Despite current recommendations, data suggest that levels of disclosure of HIV status to HIV-infected children receiving care in resource-limited settings are very low. Few studies describe the disclosure process for children in these settings, particularly the motivators, antecedent goals, and immediate outcomes of disclosure to HIV-infected children. This study examined caregivers' perception of the disclosure concept prior to disclosure, their motivation towards or away from disclosure, and their short- and long-term intentions for disclosure to their HIV-infected children.

**Methods:**

In-depth interviews were conducted with primary caregivers of 40 HIV-infected children (ages 5–15 years) who were receiving HIV care but did not know their HIV status.

**Results:**

Caregivers of HIV-infected children mainly perceived disclosure as a single event rather than a process of gradual delivery of information about the child's illness. They viewed disclosure as potentially beneficial both to children and themselves, as well as an opportunity to explain the parents' role in the transmission of HIV to the children. Caregivers desired to personally conduct the disclosure; however, most reported being over-whelmed with fear of negative outcomes and revealed a lack of self-efficacy towards managing the disclosure process. Consequently, most cope by deception to avoid or delay disclosure until they perceive their own readiness to disclose.

**Conclusions:**

Interventions for HIV disclosure should consider that caregivers may desire to be directly responsible for disclosure to children under their care. They, however, need to be empowered with practical skills to recognize opportunities to initiate the disclosure process early, as well as supported to manage it in a phased, developmentally appropriate manner. The potential role for peer counselors in the disclosure process deserves further study.

## Introduction

Globally, paediatric HIV infection continues to be a major problem. Approximately 3.3 million children younger than 15 years are living with HIV, with 2.9 million of them in sub-Saharan Africa [1]. In Uganda, just over 7% of the adult population and about 1% of children less than 5 years are HIV-infected [2]. There has been considerable progress in recent years towards making comprehensive HIV treatment accessible to children in Uganda; paediatric antiretroviral therapy (ART) services have been scaled up to over 300 health facilities throughout the country. With increasing availability of accessible, effective combination ART, paediatric HIV has been transformed from a rapidly fatal infection to a chronic disease requiring complex lifelong treatment and support [3].

The increasing survival of perinatally HIV-infected children into adolescence and adulthood has brought to the fore new and important challenges relating to adherence to long-term treatment, as well as development issues including, among many, peer relationships, puberty, and sexuality [4], [5]. The issue of HIV status disclosure - whether, when, and how to inform HIV-infected children about their HIV status - has gained increased attention in recent years [6], [7].

Disclosure of HIV status is one of the most complex challenges facing individuals who live with HIV/AIDS. It entails communication about a highly stigmatized, life-threatening, transmissible and, currently, still incurable infection. It is usually approached with much anxiety and fear of negative consequences, notably stigmatization and discrimination, and is often avoided altogether [8]. Although unintended disclosure may occur, HIV-infected adults typically make a choice to disclose their status to others. The motivation to disclose and the immediate consequences of disclosure are viewed from the affected individual's viewpoint [9].

The disclosure process in paediatric HIV is more complex and less well understood. In children, the process involves consideration, by the caregiver, of the child's developmental and emotional readiness to receive the information about his/her HIV status, as well as attitudes and motivations/goals of the caregiver and/or health worker towards disclosure [9], [10]. Caregivers and healthcare workers are presented with many challenges around disclosure, including deciding what is in the child's best interest and when, why, and how much information about his/her HIV status should be shared with him/her [11].

The World Health Organization and national paediatric HIV guidelines [6], [12] recommend developmentally appropriate HIV status disclosure to adolescents and school-age children. The WHO made the strong recommendation that children of school age should be told their HIV positive status; younger children should be told their status incrementally to accommodate their cognitive skills and emotional maturity, in preparation for full disclosure [13]. Expected benefits of disclosure include enhanced access to support services, better treatment adherence, improved family communication, and better long-term health and emotional well-being for the child [14], [15]. Reports generally indicate positive outcomes associated with disclosure in children, although stigma and depression have been reported, especially in the short-term [16]-[19].

However, current data from resource-limited settings show typically low rates of disclosure to children, ranging between 10–38% [7], [18], [20]–[22]. In a recent study conducted in the Mbarara Regional Referral Hospital in Uganda, caregivers reported that only 31% of children between 5 and 17 years had received full disclosure of their HIV status [23]. A much smaller proportion (about 7%) of children was classified as receiving partial disclosure, despite being within the age range for which initiation of the disclosure process is recommended. Additionally, because disclosure of perinatal HIV is generally delayed until late childhood or adolescence, children usually receive ART for long durations of time before the reasons for this prolonged therapy are fully discussed with them.

Reasons for these low rates of disclosure are not fully understood. In studies mainly from the developed world, caregivers cite a belief that the child is either not old enough or not ready [24], [25] or is not sufficiently mature to understand and/or cope with the diagnosis [26]; concern that if they disclosed, their child would not keep the diagnosis private; or worry that children would be exposed to ostracism and negative reactions from community and family [24]–[26]. Some HIV-infected mothers have reported the concern that their child will be angry with them for transmitting the virus [25]. Further research is needed to understand the disclosure process in low resource settings.

Moreover, few interventions to support disclosure are available and are limited to developed settings. Further data is needed to develop effective, locally appropriate HIV disclosure interventions that can be used to facilitate and support effective and beneficial disclosure in developing settings. Such interventions require an understanding of the disclosure process, including factors that affect the likelihood and the outcomes of disclosure, as well as the drivers of caregivers' decision-making towards disclosure (or concealment) of HIV status to children under their care [9]. This study examines caregivers' perception of the disclosure concept prior to disclosure, their motivation towards or away from disclosure, and their short- and long-term intentions for disclosure to their HIV-infected children in a rural Ugandan population.

## Methods

### Ethical statement

This study was approved by the Mbarara University Institutional Research Ethics Committee (MUST-IREC) and the Uganda National Council for Science and Technology. Written, informed consent was obtained from all caregivers

### Overview

In-depth interviews were conducted with the primary caregivers of HIV-infected children (ages 5–15 years) who were receiving HIV care but did not know their HIV status.

### Study setting

The study was conducted in the Paediatric HIV Clinic of the Mbarara Regional Referral Hospital, Mbarara, Uganda, located about 250 km southwest of Kampala. The hospital is a tertiary centre serving the predominantly rural population of southwestern Uganda and also serves as a primary health care facility for the population of Mbarara Municipality (approximately 100,000 people). The Paediatric HIV Clinic began providing ART in 2002 and has treated over 1,000 children cumulatively. Approximately 700 children are currently registered for comprehensive HIV care in the clinic. The children have access to free care and treatment, including ART, and the quality of medical care is considerably higher than that available elsewhere in the region. Counseling for ART adherence is offered routinely to caregivers; however, no specific training is given to caregivers to assist them with disclosure.

### Participants

The study enrolled adults who identified themselves as the current primary caregivers of children receiving HIV care in the hospital. A primary caregiver was defined as an adult, living in the same household as the child in question, who was ordinarily responsible for supervising the care of the child in the home and for bringing the child to hospital for his/her regular clinic visits. Caregivers were eligible for the study if they were above 18 years of age, had a child between 5 and 15 years in the clinic, and, to the caregiver's knowledge, disclosure of the child's HIV status had not taken place. Participants were enrolled consecutively during clinic visits. Interviews were reviewed continually until theme saturation was achieved [27]. No selection was made based on age or gender. Institutionalized children and those in boarding school were excluded, as the circumstances for disclosure likely differ greatly from those of children living in households.

### Interviews

Individual in-depth interviews were conducted, by a research assistant trained in qualitative interview methods, in a closed room away from other clinic patients and the children, to ensure privacy and avoid unplanned disclosure to the children. According to the individual subject's choice, interviews were conducted in English or Runyankore (the main local dialect in the region). With permission, all interviews were digitally recorded and later transcribed. Runyankore transcripts were translated into English post-hoc. Each caregiver was interviewed one time for 50–60 minutes on average.

Interviews began with structured questions on individual characteristics (e.g., demographics, time since diagnosis, educational levels of both caregiver and child), family characteristics (e.g., relationship of caregiver to child, marital status, child's orphan status) and the health and HIV status of the caregiver. An interview guide was developed using a *priori* themes that were anticipated based on a review of the literature and consideration of what the study team considered would emerge based on their experience interacting with HIV-infected children and their caregivers. The interviewer started the interview with open-ended questions in four main areas. First, they were asked for their personal experiences caring for HIV-infected children. Second, caregivers were asked questions to explore their perception of disclosure to children both in terms of the content and scope of the disclosure conversation. They were asked whether and when children should be told their HIV status, how much they should be told, how they should be told, and by whom. The interviewer then defined four main components as essential elements of full disclosure: 1) naming the disease (using the label HIV/AIDS or its equivalent in local dialect), 2) discussing the consequences and implications of having HIV infection, 3) discussing the origin/source of the child's HIV infection and 4) discussing the fact that the child's HIV infection can be transmitted to others. Caregivers were then asked to discuss if they would find aspects of disclosure easy or difficult. They were also asked their thoughts on potential mediating factors that may facilitate or hinder successful disclosure. Third, the interviewer asked caregivers to discuss both benefits and perceived potential harms from disclosing to the children. Finally, information was also collected on the caregivers' specific intentions regarding disclosure to children in their care, including whether and when they intended to disclose, and their personal goals for any disclosure.

### Data analysis

Data were analysed using directed content analysis [28]. Recorded interviews were transcribed and, if needed, translated directly into English by the primary interviewer. The lead investigator (JK) reviewed the transcripts for accuracy by playing back the recordings while reading through the transcripts. Thematic category construction methods were used to inductively analyze and represent the data [29]. The content analysis proceeded in three phases. In the first phase, two analysts (JK, EM) reviewed the transcripts and created codes based on topics that arose frequently and/or had relevance to perceptions, motivations, or intentions around disclosure. A preliminary codebook was developed based on 20% of the interviews and refined on review of another 20% of interviews. Key definitions of the codes were then generated and further modifications were conducted iteratively after applying the codes to the remainder of the interviews. Once the codebook was complete, the analysts conducted open coding, in which they applied a code from the codebook to a block of text to indicate the primary theme or idea being expressed. During the second phase the two analysts grouped similar codes and identified relationships between codes to identify emerging patterns of experience expressed in the data. In the third phase, summary coding, the investigative team (JK, EM, and JH) identified the central themes that emerged from analysis and separated them from outliers, to summarize the key findings across all interviews.

## Results

### Description of participants

A summary of the main characteristics of the participants is presented in Table 1. Overall, 44 eligible caregivers were approached to participate. Four caregivers declined because of time constraints. Forty caregivers were interviewed, 33 of whom were the HIV-infected biological parents (32 mothers and one father) of the children for whom they cared. The rest were HIV-uninfected or untested. All except one were females, and 31 of them were receiving ART. Most HIV-infected caregivers reported having disclosed their own status to someone in their family or community. The majority of children were between 5 and 10 years, and more than 75% were receiving ART at the time of the study.

**Table 1:** Characteristics of caregivers and children (n = 40).

Characteristic	Frequency	Percent
Caregiver's age (years)		
21–30	10	25.0%
31–40	20	50.0%
41–50	6	15.0%
51–60	3	7.5%
61–70	1	2.5%
Caregiver's sex		
Male	1	2.50%
Female	39	97.5%
Caregiver's relationship to the child		
Mother	32	80.0%
Father	1	2.5%
Grandmother	3	7.5%
Aunt	4	10.0%
Highest education level attained		
No school	3	7.5%
Primary school	28	70.0%
Secondary school	7	17.5%
Tertiary education	2	5.0%
Caregiver's HIV status		
Infected	33	82.5%
Uninfected or untested	7	17.5%
Caregiver on HAART		
Yes	31	77.5%
No	9	22.5%
Child's age (years)		
5–7	16	40.0%
8–10	17	42.5%
11–15	7	17.5%
Child's sex		
Male	20	50.0%
Female	20	50.0%
Child's age at diagnosis (years)		
0–0.9	3	7.5%
1–4.9	24	60.0%
5–9.9	12	30.0%
10–14.9	1	2.5%
Child on HAART		
Yes	31	77.5%
No	9	22.5%

HAART  =  highly active antiretroviral therapy.

### Key themes

Five major themes were identified (Table 2): 1) Caregiver understanding of the concept of HIV disclosure, 2) Caregiver attitudes, motivations, and goals for disclosure, 3) Avoiding disclosure, 4) Coping before disclosure through deception, and 5) Intentions for disclosure.

**Table 2:** Summary of categories/themes.

Caregiver understanding of the concept of HIV disclosure
Telling the truth
Complete disclosure; important components of full disclosure
Caregiver motivations for disclosure
The child should know
Benefits of disclosing
The caregiver has a responsibility to disclose
Avoiding disclosure
Fear of negative outcomes
Feeling unable to disclose
Coping before disclosure through deception
Intentions for disclosure

### Caregiver understanding of the concept of HIV disclosure

Before hearing the interviewer's definition of the components of full disclosure, caregivers emphasized two points about their understanding of the concept: 1) openness in telling the truth about the disease and 2) the importance of complete disclosure, meaning the source of the child's infection, the health implications of HIV, and potential to transmit the infection to others.

#### Telling the truth

Most of the caregivers discussed disclosure as a “truth-telling” event, primarily focused on naming the disease. It involved being honest and open, for the first time, to the child about his/her HIV status and specifically using the terms HIV or AIDS.

*“…it means telling the truth about the disease… It means you to be open and telling her that you are HIV-infected” (37-year-old mother of 10-year-old girl)*

Many caregivers discussed this truth telling as a single event that could not be drawn out into more than a single disclosure conversation. Thus, one caregiver stated:

*“Once you have prepared yourself to disclose to the child you have to say each and everything. You tell the child that she has HIV, and she got it from me. Because if you do not mention that then what will you say she is suffering from?” (35-year-old aunt of 10-year-old girl)*

#### Importance of complete disclosure

Caregivers discussed what they felt were critical components of complete disclosure. Three elements emerged, namely discussing the source of the child's infection, health implications of having HIV, and potential to transmit the infection to others.

A significant proportion of caregivers emphasized the importance of telling the child where and how he/she was infected with HIV. They recognised that this would mean inevitable disclosure of their own status. They felt, however, that they needed to dispel confusion in the child's mind since the child may only know HIV to be sexually transmitted. For some caregivers this discussion also needed to include a clarification that the transmission of HIV from the mother was unintentional.

*“I understand that you should tell the child the truth.... and you explain to him in detail how she got the disease. Tell her that you got it from me. Tell her that me as a parent I did not intend this to happen.” (32-year-old mother of 8-year-old girl)*

Many caregivers included a discussion of the long-term consequences of HIV to the child as a necessary component of disclosure.

*“You have to tell her that the disease you have is not curable, it will not go away. You will have to take medicine constantly. You will have to continue taking it, and never interrupt it. If you do that you will die.” (33-year-old mother of 9-year-old boy)*

The majority of caregivers discussed the importance of emphasizing to the child that they could transmit HIV to others through some forms of contact in the course of regular activities, like play for young children or through sexual activity in older children. As such, including this component in the disclosure package was justified as necessary to protect the child's contacts from accidental exposure to HIV. In some cases, this emphasis was used as a means for controlling the child's behaviour around sexuality.

*“Tell him that once you are of age, be careful and don't engage yourself in acts of sexual immorality. The most important thing the child should understand is not to have sexual intercourse. Other things will be told to him along the way.” (38-year-old father of 13-year-old boy)*

### Caregiver motivations for disclosure

Caregivers were unequivocal in their affirmation that children should be told that they have HIV. The three main sub-themes around their motivations for such disclosure included 1) the child should know, 2) benefits for the care of the child, and 3) the caregiver's responsibility to disclose.

#### The child should know

Caregivers generally felt that HIV-infected children needed to know their HIV status to make sense of whatever was going on in their lives, especially in terms of their health. They felt that the child has a right to this explanation.

*“Yes, it is right to tell them. When the child is with other children, and they keep on teasing him because they know his mother had HIV, and they know that he has HIV and for him he doesn't know… Once you tell him and he knows, even if he gets annoyed he still knows what he is suffering from.” (26-year-old aunt of 11-year-old boy)*

#### Benefits of disclosing

Caregivers perceived a range of potential benefits in the HIV-infected child knowing his/her status. As the caregivers had not yet disclosed, these represented their beliefs rather than experiences of the benefits of disclosure. The main benefits of disclosing to an HIV-infected child that caregivers discussed included health benefits and protection of others.

Most caregivers hoped that disclosure would motivate the child to take his/her ART consistently and take more personal responsibility for his/her own care generally. Responses emphasized caregivers' faith in ART, and their acceptance that consistent adherence would be central to the child's survival and quality of life. As such, many caregivers focused on the incurable, and ultimately fatal, nature of untreated HIV/AIDS. They anticipated that this would motivate the child to be adherent to medication.

*“The child should understand that if he stops taking his medicine he will die. You should tell the child that HIV has no cure. He has to continue taking his medicine and if he ever stops, he will die.” (33-year-old mother of 9-year-old girl)*

Getting children to understand this point was especially important for most caregivers and in some cases could lead to mutual support for adherence in both the child and caregiver.

*“Once you have told the child, he will know that he needs to take his drugs. And let's say he is at school and he doesn't want his fellow children to see him swallowing the medicine, he may decide not to take it. But once you have disclosed to him, he will try by all means and make sure he swallows the medicine.” (26-year-old aunt of 11-year-old boy)*

*“The benefit is that the child will remind himself to take medicine and he will also remind you so you find that his health and mine continues to be good because we keep taking medicine together.” (26-year-old mother of 6-year-old girl)*

For some, this expectation of better adherence and self-care was founded in prior experience with other children.

*“The benefit is there; for example like my [other] daughter, before I told her I would give her medicine to take and instead she would hide it and throw it away and she would fall sick again. After disclosing to her I saw a big change, she now takes her medicine regularly and no longer falls sick. Now when it is time for taking medicine she takes it without me reminding her.” (35-year-old mother of 9-year-old girl)*

Some caregivers discussed how disclosure will allow a child to take deliberate actions to protect his/her playmates and potential sexual partners, from accidental exposure to HIV.

*“…the child should be told because if not told she may spread the HIV to other children, but if your child knows, if she uses a razor she throws it away so that others do not get in contact with it.” (26-year-old mother of 6-year-old girl)*

#### The caregiver's responsibility to disclose

Most caregivers described disclosure as a responsibility the caregiver holds to the child. They were often preoccupied by feelings of this responsibility and the need to account to their children who, in most cases, were vertically infected. The desire to tell the child before the caregiver dies, as well as before other people tell the child, was frequently cited.

*“I want to tell him because I do not know how long I am going to live. I may die before him…, so that is why I want to tell him so that even when I am already gone he knows that I am like this; Mum told me. …So that I do not leave him in the dark. His friends might tell him and he would be like, 'my mother who gave birth to me, why could she not tell me this? '” (30-year-old mother of 12-year-old boy)*

### Avoiding disclosure

Even though caregivers had strong desires to disclose, they had not yet embarked on the disclosure process. Analysis identified three main categories of reasons for avoiding disclosure, namely fear of negative outcomes, and a lack of self-efficacy.

#### Negative outcomes

Three sub-themes capture the most common harmful outcomes that caregivers feared might result from disclosure: the child's negative emotional reactions, anticipated stigma and discrimination, and harm to the parent-child relationship.

Most caregivers were very concerned that disclosure would lead to the child experiencing sadness, shame, hating himself, despair, hopelessness, social withdrawal, shock, collapse, and even self-harm and suicide.

*“Some children I have heard who were disclosed to, some took poison after knowing. Some children lose hope and stop taking medicine; that after all they are going to die.” (34-year-old mother of 8-year-old girl)*

Children receive negative messages in the community about HIV/AIDS, such as frightening media campaigns, and in some cases, children had negative, personal experiences, including loss of a parent. Caregivers therefore expressed concern that a child would have a very poor response to disclosure. For example:

*“…especially if the child has somehow grown up. He goes to school and has learnt about HIV/AIDS and its effects. He has learnt that AIDS has no cure, it kills, and once the child already knows this information and you tell him that you have HIV, he can collapse. Some children have even hung themselves after being told… So it is very dangerous.” (30-year-old aunt of 10-year-old boy)*

Such feelings were not considered irrational or unreasonable; caregivers extrapolated them from adult experiences with first learning the diagnosis.

*“…it could be harmful to tell the child in that once he knows he can even run mad… Even an old person, when they go for testing and they are told that they have HIV/AIDS; some can even get a stroke…” (27-year-old mother of 6-year-old boy)*

Caregivers feared that the child and the family could be discriminated by the community, especially if the child disclosed intentionally or inadvertently to his/her friends, playmates, and/or schoolmates. This fear was commonly cited as a reason for avoiding disclosure.

*“They are supposed to be told but to me I think they should be told when you see that the child understands what you are going to tell him, whether it is bad and he will not take it as a usual thing, and he goes spreading it around everywhere that he has HIV” (28-year-old mother of 12-year-old child)*

Many caregivers, especially biological parents, expressed feelings of guilt for infecting their children and feared that the disclosure could harm the parent-child relationship, in some cases with disastrous consequences.

*“Telling the child that he got the disease from the parents, that is hard. That is something that usually annoys children. For example, back in our village there is a girl whose mother died of HIV. I think she might have been told, perhaps by the mother [that she has HIV]. Now the girl is in high school but she said she will hate her mother until she dies. You see, when you tell the child that he has HIV, inevitably, you have to tell him that you got it from me. It may not go well…” (Mother of 6-year-old child)*

*“It is hard for me to tell the child that he got the disease from me because he might get annoyed with me and blame me for his status. It is difficult because how do you tell the child that I was promiscuous, that your father was promiscuous, that is how we got the disease? That is very hard to tell the child, there is no way I can explain it to you. This child does not know things of mature people…” (Mother of 7-year-old child)*

#### Feeling unable to disclose

When asked which aspects of disclosure they would find easy or difficult, caregivers' responses indicated they did not feel they had the skills necessary for handling the disclosure process and dealing with anticipated adverse outcomes. They felt that disclosure was a difficult undertaking; the anticipated adverse outcomes easily overrode any desired benefits from disclosure. These concerns were expressed across all aspects of disclosure, but particularly around naming the disease and discussing consequences of having HIV.

*“Right now I am not at rest because she keeps asking me why she takes medicines… It would be good to tell her but where do you begin? You cannot tell her direct that you [the child] have HIV. I would tell her that you need to take medicine and it is important for your health, but it is very hard for me to tell her that you have HIV.*

*I will try to tell her myself but if my heart fails then I will bring her to the doctors and they disclose to her. For them, they are experts” (34-year-old mother of 10-year-old girl)*

### Coping before disclosure through deception

Caregivers were frequently presented with opportunities to begin disclosure; children often questioned why they needed to take regular medication, or occasionally, directly asked if they had HIV. Because of the above-noted fears, however, caregivers often created stories to tell HIV-infected children to avoid disclosure. Significantly, the majority spoke about these lies in the first person, meaning that they themselves had misled their children when confronted by questions about the child's HIV status or why the child was always taking medication. In some cases, such lies were told on a sustained basis and were expected to continue until disclosure finally happened. Caregivers suggested that this approach was justified if the child was too young or otherwise not ready for disclosure.

*“When they are still young people deceive them that they have cough, or he might have a wound and I tell him that you are taking medicine because of this wound; and you keep deceiving him like that until he is of age and has grown to be told the real truth.” (29-year-old mother of 6-year-old boy)*

The kinds of stories give some insight into the caregivers' fears regarding disclosure. Their purpose could be either concealment (e.g., sustaining the child's ignorance of his status) or denial (e.g., caregivers' own rejection of the reality of the HIV status). Importantly, some of stories would predictably make it more difficult for the caregivers to eventually disclose when the time comes. Thus:

*“You tell the child that the medicine you are taking is not for HIV. The child might ask you that do I have the virus and you tell the children that no, you do not have it. HIV/AIDS is only contracted through sexual intercourse, and for you where would you get it from? (27-year-old mother of 6-year-old girl)*

### Intentions for disclosure

All caregivers stated that they had intentions to disclose to their children in the future. The appropriate timing of disclosure was discussed mainly in terms of the child's age; the majority of caregivers intended to disclose when the children were between 12–15 years. When asked who they thought would be the best person to disclose to the child, most responded themselves, thus confirming their sense of personal responsibility to their children.

*“I would do it myself because it is the right thing to do.” (28-year old mother of 6-year-old boy)*

*“I think I will do it myself because the child will not know whether the other person is telling the truth or lying. But once you tell her yourself she will know that what my mother is telling me is the truth.” (34-year-old mother of 10-year-old girl)*

On the other hand, responses also revealed significant anxiety among some caregivers about their ability to handle the disclosure process. They wished for substantial support through the process, mostly from counsellors and health workers. These caregivers hoped that professional support would help them deal with any questions the children might ask, as well as difficult emotional reactions. Some caregivers also mentioned that getting support from peers and other children living with HIV/AIDS might be beneficial.

*“You know, I have a friend in the neighbourhood. When she was going to disclose to her daughter she called me and I supported her. She recently asked me if I had disclosed to my child. I said, “No”. She said to me “whenever you plan to disclose to her, call me and we shall counsel her together. Don't you recall how it went for me?” (28-year-old mother of 6-year-old boy)*

## Discussion

In this study of caregivers in a rural Ugandan setting, we found that most perceived disclosure as a single momentous event of truth-telling that is usually preceded by a period of sustained avoidance and deliberate deception. Caregivers generally viewed disclosure as potentially beneficial to children and expressed a desire to derive a wide range of anticipated benefits, particularly improved medication adherence and better self-care, for their children through a properly timed and managed disclosure. They also viewed it as an opportunity and framework for accounting to the children for their own responsibility in the children's infection. However, most were preoccupied with fear of negative outcomes and did not feel able to disclose well. It was therefore easier for them to avoid disclosure even when presented with clear opportunities to tell.

Because children typically receive ART for a long time prior to disclosure and must develop sufficiently to understand their diagnosis, unique challenges arise that are best handled through a gradual disclosure process. World Health Organization and national guidelines recommend that disclosure consist of a gradual process in which information is delivered in a graded manner appropriate to the child's development. The child is given some, but not all, the information about their illness. The child may be told that he/she has a disease that is described in a way that is consistent with HIV/AIDS, but the disease not necessarily mentioned by name [10], [30].

Caregivers in this study, however, perceived disclosure in a way similar to the post-test disclosure one would get with a first test for HIV. This finding reveals a lack of understanding of the concept of partial disclosure and suggests that children might be brought very quickly from a position of no disclosure to full disclosure. This phenomenon has been reported before; a qualitative study of 12 children in the Democratic Republic of the Congo who had received disclosure found that almost all children interviewed reported receiving their disclosure information through a single conversation with neither preparatory nor follow-on discussion after the event [19]. This approach is unlikely to be satisfactory, as it requires the child to absorb too much information, including reconciling the new knowledge with prior misinformation. Without the benefit of follow-on discussions to address questions and concerns, children could develop feelings of confusion, isolation and depression. Nonetheless, most caregivers independently described their understanding of full disclosure appropriately, even before hearing the interviewer's definition of essential components. This finding suggests that they may not need more counseling on the components of disclosure, but more on how to do it as a process.

We found that caregivers had important positive goals for disclosure including improved adherence to medication, better parent-child relationship, and community protection. Disclosure was, however, avoided because of a range of fears. Fears of negative emotional reactions by the child were similar to what has been reported in other studies [15], [22], [31], [32]. These fears are perhaps not entirely unexpected. The vast majority of caregivers interviewed were themselves HIV-infected and had real-life experience going through the process of testing and learning for the first time that they were HIV-infected. This experience might have informed their expectation that receiving news of their HIV status would be distressing for the child.

Fears of negative emotional reactions are reinforced by a fatalistic view of HIV/AIDS in the community, media, and health education programs in school. As reported in other studies [33], [34], caregivers feared that children would live in constant fear of dying once they were told they had HIV. However, such fears of adverse outcomes may be unduly exaggerated. Data from studies of the psychological impact of disclosure in children generally suggest that although some may experience some negative reactions initially, these are usually followed by more positive feelings of relief and empowerment [17], [19], [35]. Overall rates of depression, emotional and behavioural problems, for instance, are not higher among children who have been informed of their HIV status [26], [34], [36]. Helping parents understand this fact early may help mitigate these fears and get them to feel more positively about the prospect of disclosure.

Previous studies have reported that disclosure may be especially difficult for HIV-infected biological parents of HIV infected children [37], [38], primarily due to feelings of guilt and fear of blame [25]. This study confirms this finding, but also suggests that concern about distressing the child, particularly for parents who may have experienced significant emotional distress following post-test disclosure, may play a significant role.

One fear frequently cited as a reason for delayed disclosure in previous studies is that HIV-infected caregivers fear revealing their own HIV status, and the attendant concern that the child might not be able to keep the diagnosis a secret [20], [22], [35]. Our data concurs, partly, with this finding. However, even caregivers who are open about their own HIV status can have difficulties disclosing their child's HIV status. In this study, a significant number of HIV-infected parents reported having disclosed their own status within their family and community and in some cases to the HIV-infected child in question. For these parents, at least, the delay to disclose may be more likely due to a genuine desire to protect the child from distress rather than a need to preserve secrecy of their own HIV status.

Caregivers are often presented with opportunities to begin the disclosure process, mostly when children begin questioning the need for taking regular medication. However, as a strategy to cope with fear they may deflect questions about HIV. This practice may be more pervasive than has previously been reported [10]. In our study, almost all caregivers indicated that they had told their children a lie when confronted by questions about the child's HIV status. Their justification of it and the expectation that they would continue until they felt ready for disclosure highlights the fact that, for many caregivers, pretense is a major strategy for coping through the pre-disclosure period. Unfortunately, some of the lies might make future disclosure more difficult. Interventions to support caregivers through disclosure should aim to provide viable alternative strategies for managing the pre-disclosure period and support caregivers through a managed process of partial disclosure.

Ultimately, whether and when disclosure happens will depend not only on caregivers' goals and desired outcomes, but also on their ability to act on them. Dematteo and others have argued that, for disclosure of the child's HIV diagnosis to occur, adult caregivers have first to trust in their own readiness and competency to disclose [39]. Our data revealed a lack of self-efficacy among caregivers; most felt that they lacked the skill to adequately communicate with their children about their HIV status and to deal with the anticipated negative reactions. Similar to other studies, caregivers frequently anticipated a need, and expressed a desire, for professional support when the time for disclosure comes [19], [33], [40], [41].

Despite concerns about lack of ability to act independently, most caregivers still prefer that the parent or primary caregiver should be the one to disclose to children under their care. They perceived disclosure as a responsibility the caregiver holds to the child, and they see an opportunity to be accountable to their children and to foster a closer relationship of trust. Interventions to promote disclosure in this population should recognize caregivers' desire to personally conduct the disclosure process and offer support to help them overcome the barriers that hinder them from acting on this desire.

Considering caregivers' stated desire for guidance and support by health workers through the disclosure process, it is important that health workers are trained and supported with appropriate guidelines. Typically, this type of care is not routinely available in settings like this one. Even though most HIV-infected caregivers had disclosed their own status within their family or community following encouragement by health workers, they had received no specific support through the process. The suggestion of a possible role for peer counselling for the caregiver in preparation for disclosure, and the caregiver/child dyad during and after disclosure, offers potentially interesting implications for programs to support disclosure in this setting. Peer counsellors have been used to support HIV programs in African settings, particularly HIV prevention and treatment programs, with some success [42], [43].

A major strength of this study is the depth of the interviews, which explored not only the caregivers' conceptualization of disclosure, but also their motivation, intentions, and barriers with disclosure. The study focused on caregivers who had not disclosed and thus allowed an exploration of caregivers' desires and needs prior to disclosure. This study also had some limitations. Because participants did not include caregivers who had disclosed, the study does not present comparative data that could have helped identify possible facilitators of disclosure among caregivers in similar circumstances. However, the exclusion of caregivers who had disclosed was deliberate; we sought to focus on antecedent attitudes and goals of disclosure among those caregivers who might be having difficulties with disclosing. We also included caregivers whose children were relatively young and who may therefore not be developmentally ready for disclosure; however, WHO and national guidelines suggest beginning the disclosure process even at this young age. The majority of participants in this study were biological mothers; opinions of other types of caregivers may vary. Furthermore, the study was restricted to caregivers of children who were plugged into care. Further work is needed to explore perspectives of families not receiving formal care and treatment. Studies exploring the perspective of the HIV-infected child would also provide additional insight into this important issue.

## Conclusions

Caregivers of HIV-infected children mainly perceive disclosure as a single event rather than a process of gradual delivery of information about the child's illness. They anticipate a range of potential benefits to the children, as well as an opportunity for caregivers to clarify that they transmitted the virus to the child. However, most are over-whelmed with fear of negative outcomes, and reveal a lack of confidence to independently manage the disclosure process. Consequently, most did cope by deception to avoid (or delay) disclosure until they perceive readiness to disclose.

The results of this study have implications for future development of interventions to support families through disclosure-related communication. Such interventions should recognize caregivers' desire to disclose to children under their care, and the barriers that hinder them from acting on this desire. Caregivers should be empowered with practical skills to recognize opportunities to initiate the disclosure process early, and supported to manage it in a developmentally appropriate manner. Health care providers need to be equipped with skills and appropriate guidelines to be able to guide and support families towards satisfactory disclosure. The potential role for peer counselors in supporting caregivers and families through the disclosure process deserves further study.
